# Local low‐intensity vibration improves healing of muscle injury in mice

**DOI:** 10.14814/phy2.14356

**Published:** 2020-01-24

**Authors:** Thomas F. Corbiere, Timothy J. Koh

**Affiliations:** ^1^ Department of Kinesiology and Nutrition University of Illinois at Chicago Chicago IL USA

**Keywords:** cell culture, mechanical stimulation, mouse model, muscle repair, skeletal muscle injury

## Abstract

Recovery from traumatic muscle injuries is typically prolonged and incomplete. Our previous study demonstrated that whole‐body low‐intensity vibration (LIV) enhances healing in a mouse laceration model. We sought to determine whether locally applied LIV (a) improves muscle repair following injury in mice and (b) is directly transduced by cultured muscle cells, via increased IGF‐1 activity. C57BL/6J mice were subjected to laceration of the gastrocnemius muscle and were treated with LIV applied directly to the lower leg for 30 min/day or non‐LIV sham treatment (controls) for 7 or 14 days. LIV was also applied to differentiating myotubes in culture for 30 min/day for 3 or 6 days. Compared with control mice, LIV increased myofiber cross‐sectional area, diameter, and percent area of peripherally nucleated fibers, and decreased percent damaged area after 14 days of treatment. In cultured myotubes, LIV increased fusion and diameter compared with controls after 6 days of treatment. These LIV‐induced effects were associated with increased total Akt on day 7 in injured muscle and on day 3 in myotubes, whereas phosphorylated‐to‐total Akt ratio increased on day 14 in injured muscle and on day 6 in myotubes but were not associated with increased IGF‐1 levels at any time point. These changes were also associated with LIV‐induced suppression of FOXO1 and Atrogin‐1 gene expression at day 7 in injured muscle. These findings demonstrate that muscle cells can directly transduce LIV signals into increased growth and differentiation, and this effect is associated with increased Akt signaling.

## Graphical abstract

Our findings demonstrate that muscle cells can directly transduce LIV signals into increased growth and differentiation, and this effect is associated with increased Akt signaling.

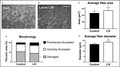

## 1 INTRODUCTION

Traumatic muscle injuries are a devastating consequence of motor vehicle accidents, limb salvage surgeries (Turner & Badylak, 2012), and military combat (Covey, 2006). They are characterized by a significant loss of tissue and inadequate healing leading to impaired muscle function, joint stiffness, and loss of mobility (Bedair et al., 2007; Menetrey, Kasemkijwattana, Fu, Moreland, & Huard, 1999; Shen, Li, Tang, Cummins, & Huard, 2005; Vaittinen, Hurme, Rantanen, & Kalimo, 2002). The regenerative capacity of muscle is highly dependent on the severity of the injury with less severe injuries able to regenerate fully and more severe injuries resulting in functional deficits due to denervation and scar formation. The only current clinical option for traumatic muscle injury is autologous muscle transfer using muscle flaps or vascularized tissue (Whiteside, 2014). These surgical procedures, however, are technically difficult, have limited success, and result in donor site morbidity (Turner & Badylak, 2012). Due to these limitations, experimental approaches in the form of tissue engineered xenotransplants made from decellularized tissue (Urciuolo & De Coppi, 2018) and cell therapy (Qazi et al., 2019) are currently being explored. There is an urgent need for new therapies that can restore functional muscle tissue following traumatic injury.

Physical therapy can be an adjunct treatment for patients with traumatic muscle injury. Skeletal muscle is known to respond to changes in mechanical loading with changes in muscle mass; resistance exercise can cause muscle hypertrophy, whereas unloading leads to atrophy (Marimuthu, Murton, & Greenhaff, 2011; Russell, 2010; Spangenburg, 2009). Standard of care for patients with less severe muscle injuries, such as sports‐related strains, involves early mobilization of the muscle (Contreras‐Munoz et al., 2017; Jarvinen, 1975). Unfortunately, patients suffering from traumatic muscle injury often have limited use of the affected limb and limited mobility due to the severity of the injury and accompanying pain. Alternate forms of physical therapy that do not require active muscle force production by the patient and that can be applied passively could overcome this obstacle to physical therapy treatment.

Vibration therapy may be a promising candidate for passively applying mechanical stimuli to muscle. To date, the majority of studies exploring the effects of vibration on skeletal muscle focus on neuromuscular outcomes related to strength and power in exercise, and a recent review of these studies shows conflicting evidence with many studies reporting that vibration increases muscle strength while others report no change (Alam, Khan, & Farooq, 2018). Many of these studies apply signals ranging from 5 g to 32 g acceleration, which can be considered high‐intensity vibration. We recently showed that low‐intensity vibration (LIV; <1 g acceleration) applied to the whole body via a vibrating plate daily to mice recovering from laceration injury increased myofiber size by day 14 compared with sham LIV, injured controls, suggesting that LIV may improve muscle repair (Corbiere, Weinheimer‐Haus, Judex, & Koh, 2017). However, much remains to be learned about the role of vibration in muscle repair as well as the mechanisms involved.

Insulin‐like growth factor 1 (IGF‐1) is a well‐known regulator of skeletal muscle mass via activation of Akt/mTOR signaling and the downregulation of myostatin signaling (Schiaffino, Dyar, Ciciliot, Blaauw, & Sandri, 2013). IGF‐1 may mediate muscle hypertrophy in models of stretch‐induced hypertrophy (Czerwinski, Martin, & Bechtel, 1994), overload‐induced hypertrophy (Matheny, Merritt, Zannikos, Farrar, & Adamo, 2009), and compensatory hypertrophy (DeVol, Rotwein, Sadow, Novakofski, & Bechtel, 1990) in rodents and transgenic mice overexpressing IGF‐1 specifically in muscle showed an increase in muscle size (Hennebry et al., 2017). In addition, two isoforms, IGF‐1Ea, a muscle‐specific isoform, and mechanogrowth factor (MGF) are upregulated in muscle after mechanical stimulation (McKoy et al., 1999; Velloso & Harridge, 2010). Furthermore, vibration of 30 Hz/4 mm displacement in combination with isometric squatting in aged men and women increased serum IGF‐1 levels (Cardinale et al.,) and vibration training in combination with isometric squatting increased MGF levels associated with increased leg extension force production (Kern et al., 2011). Because of its role as a positive regulator of hypertrophy and a negative regulator of atrophy during mechanical loading, IGF‐1 may be a mediator of LIV‐induced myofiber growth after injury.

The purpose of this study was to explore the mechanism(s) by which LIV improves muscle healing following laceration injury and if muscle cells themselves directly transducing these mechanical signals into increased growth. We hypothesized that locally applied LIV would (1) improve muscle regeneration similar to whole‐body application and (2) could be transduced by cultured myotubes, via increased levels of IGF‐1 and associated downstream signaling.

## 2 METHODS AND MATERIALS

### 2.1 Animals

C57BL/6J mice were obtained from The Jackson Laboratory and housed individually in a pathogen‐free, barrier facility with a 12‐hr light/dark cycle at a constant temperature and humidity. Experiments were performed on male mice 11–13 weeks old. Following traumatic muscle injury, mice were randomly assigned to the LIV treatment group or the non‐LIV sham control group, starting on the day of wounding. All procedures involving animals were approved by the Animal Care Committee at the University of Illinois at Chicago.

### 2.2 Muscle injury

Bilateral laceration of the gastrocnemius muscle was used as a model of traumatic injury and was performed as previously described (Novak, Weinheimer‐Haus, & Koh, 2014). Briefly, mice were anesthetized with isoflurane, and a longitudinal incision was made through the skin on the posterior of the leg to expose the gastrocnemius muscle. A scalpel was used to lacerate the lateral gastrocnemius transversely at its widest point from the central neurovascular complex (taking care to preserve the integrity of this complex) to the lateral edge of the muscle. The laceration goes through the entire thickness of the mid‐belly of the muscle which is approximately 2–3 mm thick. The skin was closed, and the procedure was repeated on the contralateral leg. For histological assays, gastrocnemius muscles were harvested, embedded in freezing medium, and flash frozen in 2‐methylbutane cooled on dry ice. Serial transverse 10‐μm‐thick cryosections were taken throughout the entire injured portion of the muscle. Sections with the greatest percentage of damaged, nonregenerated area were then selected for further analysis by staining with hematoxylin and eosin. For experiments involving protein and RNA analysis, muscles were stored in liquid nitrogen until processing.

### 2.3 Locally applied low‐intensity vibration

For in vivo LIV treatment, mice were anesthetized with isoflurane and placed in a supine position with their feet attached directly to the actuator of the vibration device. LIV was applied horizontally at a frequency of 90 Hz and peak acceleration of 0.2 g (amplitude <0.1 mm) for 30 min per day for either 7 or 14 days. Nonvibrated sham controls were treated identically but were not subjected to LIV. For in vitro LIV treatment, cell culture plates were vibrated with the same parameters using specially designed plate holders. For cell stimulation, LIV was applied daily for either 3 or 6 days. LIV signals were calibrated using an accelerometer attached directly to actuator of the vibration device. The combination of 90 Hz frequency and 0.2 g acceleration was chosen because these parameters have been used to improve healing following muscle injury (Corbiere et al., 2017) and ameliorate bone loss in rodents (Judex, Lei, Han, & Rubin, 2007).

### 2.4 Histology

Muscle regeneration was assessed by histological analysis as previously described (Novak et al., 2014). Regeneration was quantified in hematoxylin and eosin‐stained sections by morphological analysis on five representative images of each muscle section obtained using a Nikon Instruments Eclipse 80i microscope with a 40× objective, a DS‐Fi1 digital camera, and NIS Elements software (Nikon, Melville, NY). Images were taken within the muscle belly and care was taken to avoid extramuscular connective tissue. Fibers were identified as either centrally nucleated or peripherally nucleated with no evidence of damage. Centrally nucleated fibers likely represent both fibers that have undergone denervation and those in the process of regeneration (Grounds, 2014). Percent of total fibers that was classified as centrally or peripherally nucleated were then quantified, as were minimum fiber diameter and fiber area, using ImageJ (NIH). Damaged area was quantified by subtracting the sum of the area of all fibers from the total muscle area within the field of view.

### 2.5 ELISA

For ELISA, muscle samples were homogenized in ice‐cold buffer (40 mM Tris (pH 7.5), 1 mM EDTA, 5 mM EGTA, 0.5% Triton X‐100, 25 mM β‐glycerophosphate, 25 mM NaF, 1 mM Na3VO4, and protease inhibitor cocktail (#P8340, Sigma‐Aldrich) using a dounce homogenizer. After centrifugation, supernatants were diluted 1:4 and protein concentrations for IGF‐1 were determined by enzyme‐linked immunoassay (R&D Systems). Each sample analyzed in duplicate and a standard curve using recombinant protein was run with each ELISA plate.

### 2.6 Cell culture

C2C12 mouse myoblasts (CRL‐1772; American Type Culture Collection) were grown on tissue culture treated plates and maintained in DMEM (#10‐014‐CV, Corning) containing 10% FBS in a humidified incubator at 5% CO_2_ and 37 degrees C. All experiments were performed with cells between passage 5 and 7. For all experiments, cells were seeded at a density of 10^4^ cells/cm^2^. Cells were maintained in DMEM with 10% FBS (#26140; Gibco) until they were 90%–100% confluent (approximately 48 hr). Culture medium was then changed to DMEM with 2% horse serum (#26050; Gibco) to induce differentiation of myoblasts into myotubes. Starting immediately following the change in media, cells were treated daily with LIV for 30 min at room temperature or treated identically without LIV for the indicated time points.

### 2.7 Immunocytochemistry

Cultured cells were stained directly on culture plates. Cells were washed with cold PBS, fixed with 4% paraformaldehyde for 20 min, permeabilized and blocked in 0.1% Triton X‐100, 3% bovine serum albumin (BSA), incubated at room temperature for 1 hr with primary antibody for myosin heavy chain (MHC) (MF‐20; Developmental Studies Hybridoma Bank), washed with PBS, incubated at room temperature with FITC‐conjugated goat anti‐mouse IgG secondary antibody (#115‐095‐003, Jackson Immunoresearch), washed with PBS, and mounted in mounting medium with DAPI (Vectashield, H‐1200; Vector).

Myotube size and fusion were quantified in wells double stained for MHC (MF20) and nuclei (DAPI) by morphological analysis on five representative images of each sample obtained using a Keyence BZ‐X710 All‐in‐One Fluorescence Microscope with a 20× objective. Images were analyzed using ImageJ (NIH). Measurements were taken for total nuclei count, myotube count (# of MHC+ cells with 2+ nuclei), myotube nuclei count (# of nuclei in MHC+ cells that contain 2+ nuclei), and diameter. Diameter measurements were taken by averaging three separate measurements taken along the entire length of the myotube. Fusion index was defined as the ratio of myotube nuclei to total nuclei per field.

### 2.8 SDS‐PAGE and western blotting

For Western blotting, muscle samples were homogenized in ice‐cold buffer (40 mM Tris (pH 7.5), 1 mM EDTA, 5 mM EGTA, 0.5% Triton X‐100, 25 mM β‐glycerophosphate, 25 mM NaF, 1 mM Na3VO4, and protease inhibitor cocktail) using a dounce homogenizer. Cells were collected in the same buffer using a cell scraper. All tissue homogenates and cell lysates were centrifuged, and protein concentration was determined using Pierce 660 nm Protein Assay Kit (Thermofisher Scientific). Homogenates were prepared with equal protein concentrations and then dissolved in 4× Laemmli buffer (#1610747; BioRad) and heated at 95 degrees C for 5 min. Samples were immediately subjected to separation via SDS‐PAGE, transferred to a nitrocellulose membrane, blocked with Odyssey TBS blocking buffer (#927‐50000, LI‐COR Biosciences) for 1 hr at room temperature, incubated overnight with primary antibody (Akt, #9272; Phospho‐Akt (S473), #9271; p70S6K, #9202; Phospho‐p70S6K (T389), #9205; Cell Signaling Technology, Inc.; β‐Tubulin I, T7816, Sigma Aldrich) in blocking buffer at 4 degrees C, washed in TBS containing 0.1% Tween 20 (TBST), incubated for 30 min in secondary antibody (anti‐mouse, #925‐68070; anti‐rabbit, #925‐32211; LI‐COR) in blocking buffer at room temperature, washed in TBST, and finally imaged on the LI‐COR Odyssey CLx. Manufacturer quality control literature was used to select antibodies and a molecular weight ladder was run with each blot to verify size of intended target. Densitometry of each blot was performed using ImageJ (NIH); density of each protein measured normalized to β‐tubulin density.

### 2.9 RNA isolation, reverse transcription, and polymerase chain reaction

For RNA analysis, muscle samples were homogenized in Trizol (#15596018, Thermofisher) using stainless‐steel beads in a bead homogenizer. Cells were washed in PBS and collected in Trizol. All tissue homogenates and cell lysates were centrifuged, and the supernatants were used for RNA isolation. Briefly, chloroform was added for phase separation, the aqueous phase was removed, isopropanol was added for precipitation of RNA, washed in 75% ethanol, and reconstituted in DEPC‐treated water. Purified RNA concentration was measured using a Nanodrop 2000 (Thermofisher Scientific). Equivalent concentrations of RNA were prepared, and cDNA was reverse transcribed using the High Capacity cDNA Reverse Transcription Kit (#4368814; Applied Biosystems). mRNA expression was measured with Power SYBR Green PCR Master Mix (#4367659; Applied Biosystems) using the ViiA7 Real‐Time PCR System (Applied Biosystems). Primers are listed in Table 1. Relative ΔΔCT values were calculated by determining the difference in CT value compared with the housekeeping gene (ΔCT) and then calculating 2^(ΔCT sample‐average ΔCT control group)^.

**Table 1:** List of Primers used for Quantitative PCR

Gene	Primer
GAPDH	F: CATCACTGCCACCCAGAAGACTG R: ATGCCAGTGAGCTTCCCGTTCAG
IGF‐1	F: GTGGATGCTCTTCAGTTCGTGTG R: TCCAGTCTCCTCAGATCACAGC
IGF‐1 Ea	F: GCTTGCTCACCTTTACCAGC R: AATGTACTTCCTTCTGGGTCT
IGF‐1 Ec/MGF	F: GCTTGCTCACCTTTACCAGC R: AAATGTACTTCCTTTCCTTCTC
IGF‐1R1	F: CGGGATCTCATCAGCTTCACAG R: TCCTTGTTCGGAGGCAGGTCTA
Pax7	F: GTTCGGGAAGAAAGAGGACGAC R: GGTTCTGATTCCACATCTGAGCC
M‐Cadherin	F: AGGACGAGCATAGCTGAAGGAG R: GTCCACTTGCAGCCAGTCTTCT
MyoD	F: GCACTACAGTGGCGACTCAGAT R: TAGTAGGCGGTGTCGTAGCCAT
Myogenin	F: CCATCCAGTACATTGAGCGCCT R: CTGTGGGAGTTGCATTCACTGG
Myostatin	F: AACCTTCCCAGGACCAGGAGAA R: GGCTTCAAAATCGACCGTGAGG
FOXO1	F: CTACGAGTGGATGGTGAAGAGC R: CCAGTTCCTTCATTCTGCACTCG
FOXO3a	F: CCTACTTCAAGGATAAGGGCGAC R: GCCTTCATTCTGAACGCGCATG
Atrogin‐1	F: CTTCTCGACTGCCATCCTGGAT R: TCTTTTGGGCGATGCCACTCAG
MuRF‐1	F: TACCAAGCCTGTGGTCATCCTG R: ACGGAAACGACCTCCAGACATG

### 2.10 Statistical analyses

Values are reported as means ± standard deviation. Data were tested for homoscedasticity and those passed were compared using two‐sided *t* tests and those that did not pass were compared using the nonparametric Mann–Whitney test. Differences between groups were considered significant if *p* ≤ .05. Graphpad Prism Version 7.00 was used to generate all figures.

## 3 RESULTS

### 3.1 Locally applied LIV enhances muscle fiber regrowth following traumatic injury

**Figure 1:**
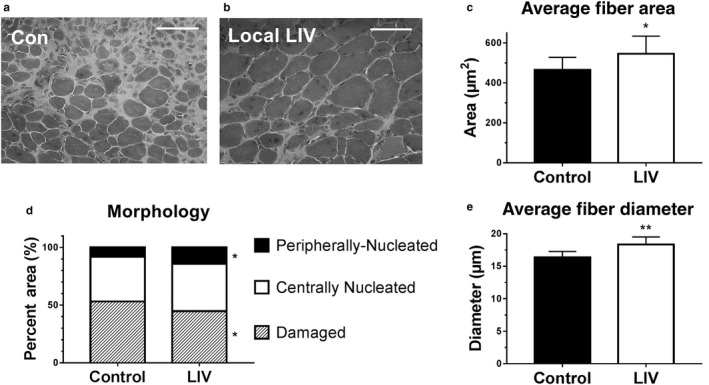
Locally applied low‐intensity vibration (LIV) enhances myofiber growth following laceration muscle injury in mice. Gastrocnemius muscles were lacerated and subjected to local LIV at 90 Hz and 0.2 g in daily bouts lasting 30 min for 14 days. Muscle fiber morphology was assessed in cryosections from the injured portion of the muscle. (a, b) Representative images of hematoxylin and eosin‐stained sections (scale bar = 50 m, 40× magnification). (c) Cross‐sectional area of myofibers, (d) percent area of injury that consists of peripherally nucleated fibers, centrally nucleated fibers, or damaged tissue, and (e) minimum diameter of myofibers was quantified in five 40x fields per muscle in hematoxylin and eosin‐stained sections. *N* = 3 replicates per group in two separate experiments for a total of *N* = 6 per group. Data are presented as mean ± *SD*. *p < .05, **p < .001

Consistent with our hypothesis, locally applied LIV treatment improved healing of lacerated gastrocnemius muscle at day 14 postinjury (Figure 1). Daily local LIV treatment increased both the mean minimum muscle fiber diameter (by 12%) and muscle fiber cross‐sectional area (by 18%) compared with sham‐treated, but injured, control mice (Figure 1). Furthermore, the area occupied by peripherally nucleated myofibers in muscle cross sections was significantly increased from 8% to 14% of total muscle area and damaged area was significantly decreased from 53% to 45% in mice treated with LIV compared with controls. The percent area of centrally nucleated myofibers was not different between LIV‐treated and control mice (Figure 1). In our previous study, whole‐body LIV treatment increased diameter by 12% and area by 34%, however, there were no significant changes in damaged area, or the area occupied by peripherally nucleated myofibers. These data indicate that locally applied LIV improves muscle healing to a similar degree, or perhaps better than, WBV.

### 3.2 Locally applied LIV does not alter IGF‐1 protein or mRNA levels in damaged muscle

**Figure 2:**
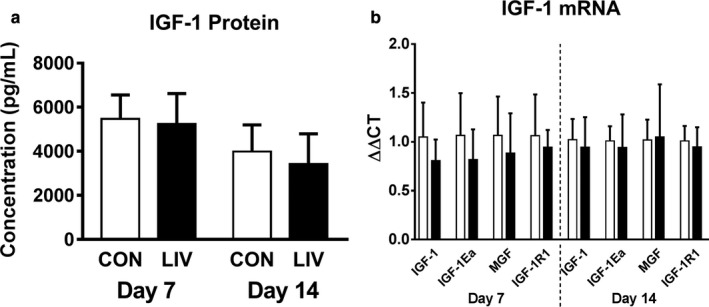
Locally applied low‐intensity vibration (LIV) does not alter IGF‐1 protein or mRNA levels following laceration muscle injury. Gastrocnemius muscles were lacerated and subjected to local LIV at 90 Hz and 0.2 g in daily bouts lasting 30 min for 7 or 14 days. Muscles were snap frozen in liquid nitrogen, and later processed for either protein or mRNA analysis. (a) Total IGF‐1 protein concentration in injured muscle at day 7 and day 14 postinjury measured by ELISA. (b) mRNA expression levels of total IGF‐1, local IGF‐1 isoforms IGF‐1Ea and MGF, and IGF‐1R1 at day 7 and 14 postinjury measured by real‐time PCR. *N* = 3 replicates per group in two separate experiments for a total of *N* = 6 per group. Data are presented as mean ± *SD*. No significant differences were observed between sham controls and LIV‐treated mice

As we previously reported that LIV increased IGF‐1 levels in skin wounds associated with improved healing (Weinheimer‐Haus, Judex, Ennis, & Koh, 2014) and IGF‐1 is a well‐known promoter of muscle growth and regeneration (Philippou, Halapas, Maridaki, & Koutsilieris, 2007), we sought to determine if the local LIV‐induced improvements in muscle healing were mediated by IGF‐1. Total IGF‐1 protein concentration was measured by ELISA and IGF‐1, IGF‐1R1, IGF‐1Ea, and MGF mRNA expression were measured by qPCR. IGF‐1R1 is the tyrosine kinase receptor that is activated by IGF‐1 and is upregulated in L6 myoblasts exposed to mechanical stimulation via cyclic stretch (Fu, Yin, Lin, Lu, & Wang, 2018) and IGF‐1Ea and MGF are individual isoforms of IGF‐1 that are known to be expressed locally in skeletal muscle in response to mechanical stimulation (McKoy et al., 1999). Local LIV treatment had no effect on total IGF‐1 protein levels or mRNA expression of total IGF‐1, IGF‐1Ea, MGF, or IGF‐1R1 after 7 or 14 days of treatment (Figure 2). These data indicate that, unlike skin wound healing, increases in IGF‐1 do not appear to mediate local LIV‐induced improvements in muscle healing.

### 3.3 Locally applied LIV may enhance Akt signaling in damaged muscle

**Figure 3:**
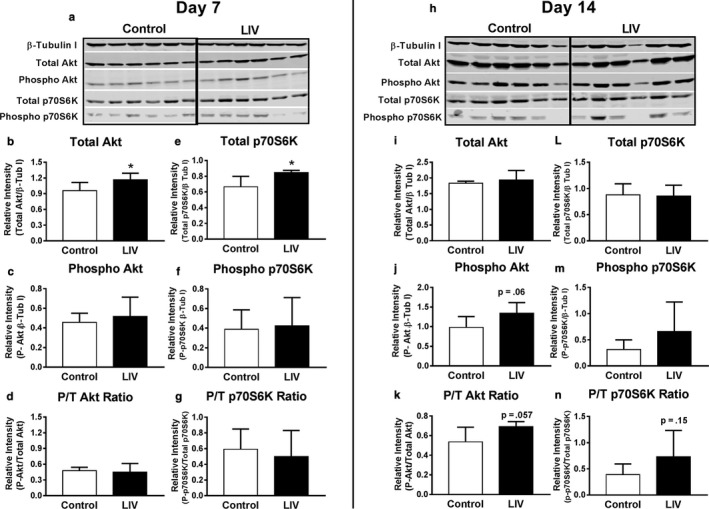
Locally applied low‐intensity vibration (LIV) increases Akt protein levels and phosphorylation following laceration muscle injury. Gastrocnemius muscles were lacerated and subjected to local LIV at 90 Hz and 0.2 g in daily bouts lasting 30 min for 7 or 14 days. Muscles were snap frozen in liquid nitrogen, and later processed for SDS‐PAGE/Western blot analysis. (a, h) Western blot images for total and phosphorylated Akt and p70S6K at day 7 and day 14 postdifferentiation. (b, i) Total Akt normalized to β‐Tubulin I. (c, j) Phosphorylated Akt normalized to β‐Tubulin I. (d, k) Phosphorylated Akt/Total Akt Ratio. (e, l) Total p70S6K normalized to β‐Tubulin I. (f, m) Phosphorylated p70S6K normalized to β‐Tubulin I. (g, n) Phosphorylated p70S6K/Total p70S6K Ratio. *N* = 3 replicates per group in two separate experiments for a total of *N* = 6 per group. All control and LIV samples run on same gel; black line inserted for clarity in separating groups. Data are presented as mean ± *SD*. *p < .05

The Akt/mTOR/p70S6K pathway is well known for playing a central role in the complex network of hypertrophic and atrophic signaling in skeletal muscle, both in response to changes in mechanical loading and following injury (Schiaffino et al., 2013). The Akt/mTOR/p70S6K pathway can be activated by IGF‐1 and although we did not find any effect of LIV on IGF‐1 levels, this pathway can also influence muscle growth via IGF‐1‐independent mechanisms (Goodman & Hornberger, 2014; Spangenburg, Le Roith, Ward, & Bodine, 2008). Thus, we sought to determine if components of the Akt/mTOR/p70S6K pathway are upregulated in response to local LIV using Western blots. At day 7 postlaceration injury, there was a significant increase in protein levels of total Akt (by 22%) and total p70S6K (by 27%) but no increase in the phosphorylated form or the ratio of phosphorylated to total protein for either molecule (Figure 3a–g). In contrast, at day 14 post‐laceration injury, there was no change in levels of total Akt or p70S6K; however, there was a trend toward an increase in phosphorylated Akt (by 36%, *p* = .06) and the phosphorylated‐to‐total Akt ratio (by 29%, *p* = .057) (Figure 3h–n). These data suggest that local LIV may first increase the signaling capacity of the Akt/p70S6K pathway at day 7 and then may increase signaling through this pathway at day 14. These changes may occur independently of mTOR as no changes in the phosphorylation of p70S6K were observed.

### 3.4 Locally applied LIV downregulates atrophy gene expression in damaged muscle

**Figure 4:**
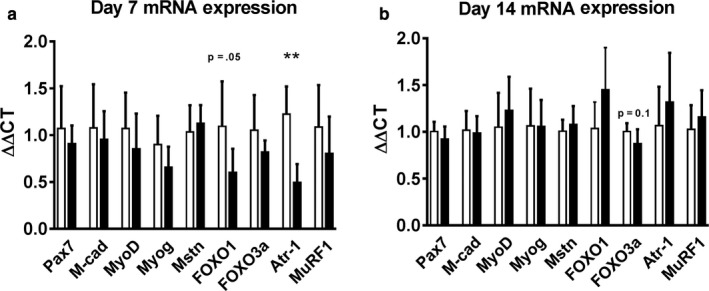
Effects of locally applied low‐intensity vibration (LIV) on expression of genes associated with myogenesis and muscle hypertrophy/atrophy following laceration muscle injury. Gastrocnemius muscles were lacerated and subjected to local LIV at 90 Hz and 0.2 g in daily bouts lasting 30 min for 7 or 14 days. Muscles were snap frozen in liquid nitrogen, and later processed for real‐time PCR. Genes measured: Pax 7, m‐cadherin (M‐cad), MyoD, Myogenin (Myog), Myostatin (Mstn), FOXO1, FOXO3a, Atrogin‐1 (Atr‐1), and Muscle Ring Finger 1 (MuRF1). (a) Day 7 and (b) Day 14 mRNA Expression. GAPDH was used as the house‐keeping gene and all samples are represented as ΔΔCT which represents the fold‐expression compared with the average of the control group. *N* = 3 replicates per group in two separate experiments for a total of *N* = 6 per group. Data are presented as mean ± *SD*. Data are presented as mean ± *SD*. * *p* < .05, **p < .01, ***p < .001

As Akt plays a central role in regulating changes in muscle mass, we also measured the mRNA expression of several markers associated with myogenesis, muscle growth, and atrophy including those for satellite cells (Pax7) myoblasts and myotubes (M‐cadherin, MyoD, and myogenin), as well as genes related to atrophy (Myostatin, FOXO1, FOXO3a, Atrogin‐1, and Muscle Ring Finger‐1). Interestingly, despite the increase in muscle fiber size, none of the myogenesis‐related genes (Pax7, M‐cadherin, MyoD, and myogenin) was upregulated in response to local LIV either at day 7 or 14 postinjury. Among the atrophy‐related genes, FOXO1 (*p* = .05) and Atrogin‐1 (*p* < .01) mRNA expression were decreased by 45% and 59%, respectively, with the application of local LIV on day 7 postinjury (Figure 4a); the others were not significantly altered. No differences were found at day 14 (Figure 4b). Interestingly, FOXO1 and Atrogin‐1 are known to be downregulated when Akt activity is upregulated (Leger et al., 2006) suggesting a possible role of these atrophy genes early in LIV‐induced muscle fiber growth following laceration injury.

### 3.5 LIV enhances growth of differentiating C2C12 cells

**Figure 5:**
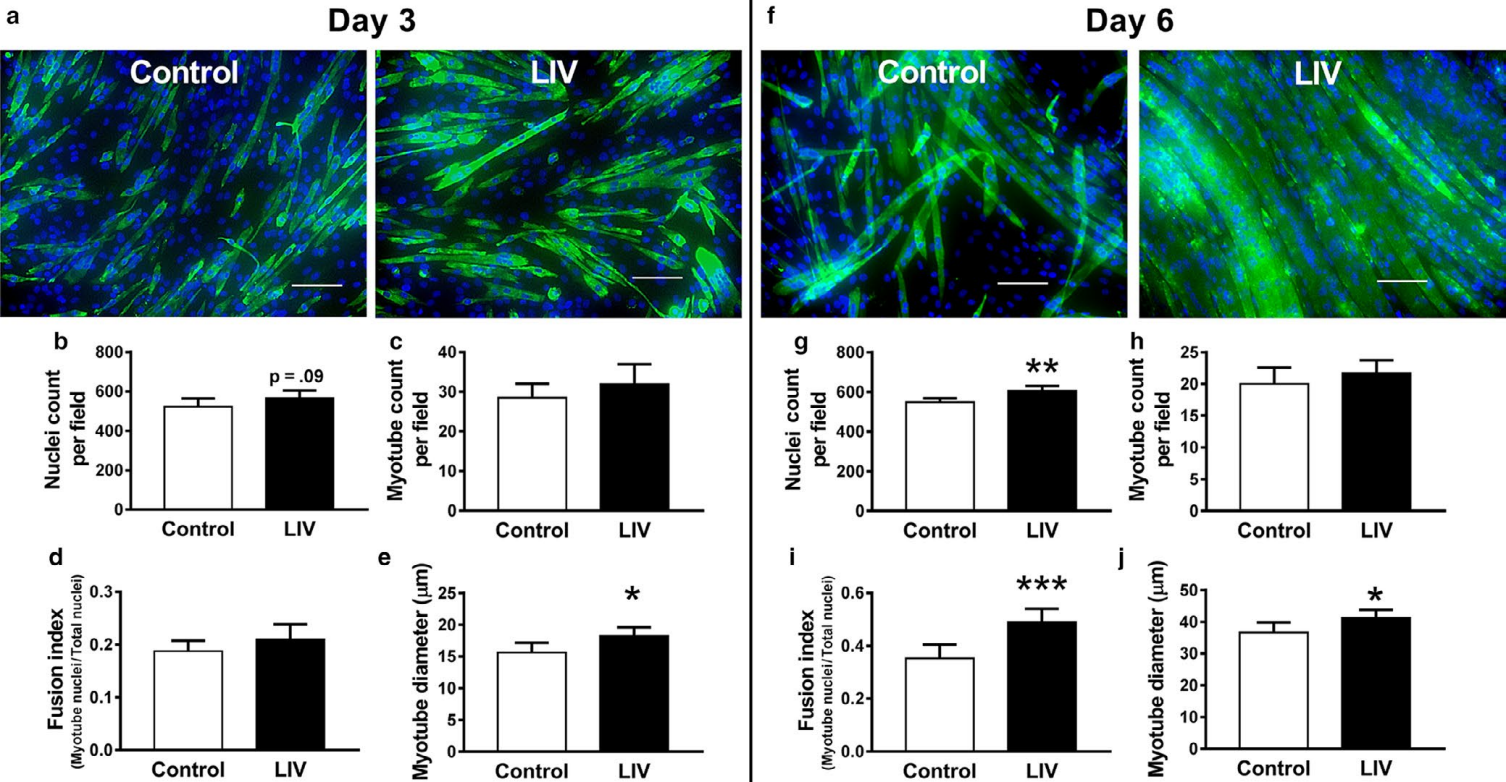
Low‐intensity vibration (LIV) increases size of C2C12 myotubes. LIV was applied to differentiating C2C12 cells at 90 Hz and 0.2 g in daily bouts lasting 30 min for either 3 or 6 days. (a, f) Representative images of C2C12 cells stained with MF‐20 antibody for myosin heavy chain (green) and nuclei stained with DAPI (blue) at day 3 and day 6 of differentiation. (b, g) Average total nuclei count per field. (c, h) Average myotube count per field. Myotubes determined as MF‐20 + cells with 2 + nuclei. (d, i) Average Fusion Index calculated by nuclei in myotubes/ total nuclei. (e, j) Myotube Diameter calculated by averaging the diameter at three locations across the length of the myotube. All values were quantified in five 20× fields per sample, *N* = 3 replicates per group in two separate experiments for a total of *N* = 6 per group. Data are presented as mean ± *SD*. *p < .05, **p < .01, ***p < .001

As locally applied LIV increases growth of muscle fibers after injury, we sought to determine if LIV signals are transduced directly by muscle cells. To accomplish this goal, we applied LIV daily to cultured C2C12 mouse muscle cells following the initiation of differentiation and measured markers of myoblast fusion into myotubes as well as myotube size at days 3 and 6. On day 3, LIV increased myotube diameter by 17% compared with control cells with no changes in total nuclei count, myotube nuclei count, or fusion index (ratio of myotube nuclei count/ total nuclei count) (Figure 5a–e). By day 6, LIV‐treated cells had a significantly greater total nuclei count (by 11%), fusion index (by 39%), and myotube diameter (by 13%). These data indicate that muscle cells are capable of directly transducing LIV signals into enhanced differentiation and growth.

### 3.6 LIV enhances Akt signaling in differentiating myocytes

**Figure 6:**
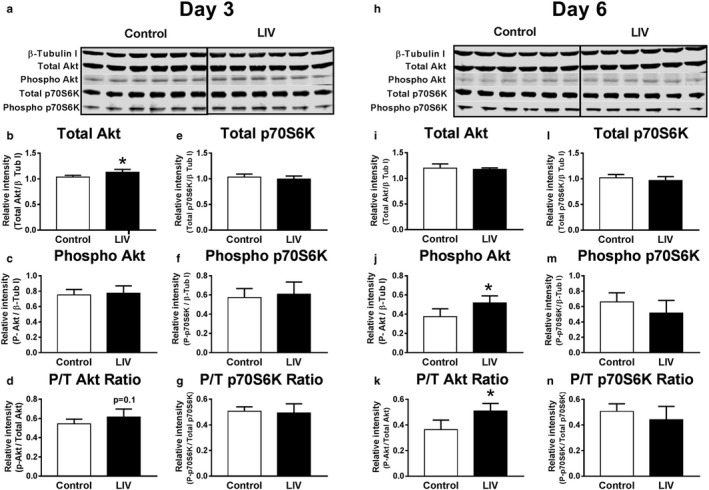
Low‐intensity vibration (LIV) increases Akt protein levels and phosphorylation in C2C12 myotubes. LIV was applied to differentiating C2C12 cells at 90 Hz and 0.2 g in daily bouts lasting 30 min for either 3 or 6 days. Cells collected and then processed for SDS‐PAGE/Western blot analysis. (a, h) Western blot images for total and phosphorylated Akt and p70S6K at day 3 and day 6 postdifferentiation. (b, i) Total Akt normalized to β‐Tubulin I. (c, j) Phosphorylated Akt normalized to β‐Tubulin I. (d, k) Phosphorylated Akt/Total Akt Ratio. (e, l) Total p70S6K normalized to β‐Tubulin I. (f, m) Phosphorylated p70S6K normalized to β‐Tubulin I. (g, n) Phosphorylated p70S6K/Total p70S6K Ratio. *N* = 3 replicates per group in two separate experiments for a total of *N* = 6 per group. All control and LIV samples run on same gel; black line inserted for clarity in separating groups. Data are presented as mean ± *SD*. *p < .05

As LIV enhanced C2C12 myotube differentiation and growth, we aimed to determine if Akt signaling might mediate this response. Cells treated with LIV had significantly greater levels of total Akt (by 10%) than control cells at day 3, whereas the ratio of phosphorylated‐to‐total Akt showed a trend toward an increase (*p* = .1) and phosphorylated Akt did not change (Figure 6a–d). At day 6, there was no difference in total Akt levels but there was a 39% increase in phosphorylated Akt and a 40% increase in phosphor/total Akt ratio in cells treated with LIV compared with controls (Figure 6h–k). No differences were observed between groups at either day 3 or 6 for total or phosphorylated p70S6K or the phospho/total ratio (Figure 6e–g,l–n). Similar to what we observed in injured muscle; these data suggest that LIV‐induced increases in myotube size might be mediated by Akt signaling in a manner that is independent of changes in p70S6K signaling.

### 3.7 LIV does not change myogenic gene expression in differentiating myocytes

**Figure 7:**
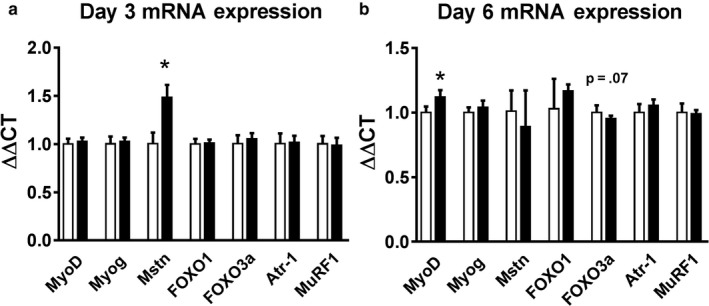
Effects of low‐intensity vibration (LIV) on C2C12 cell mRNA expression at day 3 and 6 of differentiation. LIV was applied to differentiating C2C12 cells at 90 Hz and 0.2 g in daily bouts lasting 30 min for either 3 or 6 days. Cells collected and then processed for real‐time PCR. Genes measured: MyoD, Myogenin (Myog), Myostatin (Mstn), FOXO1, FOXO3a, Atrogin‐1 (Atr‐1), and Muscle Ring Finger 1 (MuRF1). (a) Day 3 mRNA expression. (b) Day 6 mRNA expression. *N* = 3 replicates per group in two separate experiments for a total of *N* = 6 per group. Data are presented as mean ± *SD*. *p < .05

We also measured the expression of genes related to satellite cell activation, myoblast proliferation, differentiation, growth, and atrophy in myotubes. At day 3, myostatin was significantly increased with the application of LIV compared with controls (Figure 7a) which is unexpected considering myostatin is a marker associated with muscle atrophy and is known to be downregulated by Akt (Retamales et al., 2015). However, none of the other atrophy‐related genes (FOXO1, FOXO3a, Atrogin‐1, and Muscle Ring Finger‐1) that are downstream from myostatin were changed with LIV. At day 6, MyoD expression was significantly upregulated while there was a trend toward a decrease in FOXO3a expression (*p* = .07) (Figure 7b). These data suggest that LIV‐induced increases in myotube size were not strongly associated with changes in expression of genes related to myogenesis or atrophy.

## 4 DISCUSSION

Traumatic muscle injuries can result in persistent disability, as recovery from these injuries is prolonged and incomplete (Covey, 2006; Cross, Ficke, Hsu, Masini, & Wenke, 2011; Owens, Kragh, Macaitis, Svoboda, & Wenke, 2007), resulting in long‐lasting impairments of muscle and joint function (Bedair et al., 2007; Menetrey et al., 1999; Shen et al., 2005; Vaittinen et al., 2002). Thus, there is a need for therapies capable of restoring functional muscle tissue. Early mobilization of muscle following injury leads to improved regeneration (Contreras‐Munoz et al., 2017; Jarvinen, 1975), however, limited use of the affected limb, limited mobility, and pain often thwart this treatment option. LIV represents a noninvasive physical therapy strategy that circumvents these obstacles. Previously, we showed that whole‐body LIV enhances myofiber growth in a mouse laceration injury model (Corbiere et al., 2017), however, little is known about the mechanisms involved. In this study, we demonstrate that locally applied LIV improves muscle healing, and that muscle cells can directly transduce LIV signals into enhanced myofiber growth, associated with increased Akt signaling, but independent of changes in IGF‐1 levels.

Locally applied LIV induced larger myofiber size and improved muscle morphology, compared with injured, but sham‐treated controls. These improvements in muscle healing were not associated with any changes in IGF‐1 protein or mRNA expression. However, our data suggest that LIV may first increase the signaling capacity of Akt at an early time point, and then increase signaling through this pathway at a later time point. To further evaluate the pathways involved, we measured the expression of genes associated with muscle regeneration and atrophy. Notably, there were no changes in any genes associated with myogenesis; however, there were significant decreases early during repair in FOXO1 and Atrogin‐1 expression, which are associated with muscle atrophy. When activated, Akt phosphorylates FOXO1 and sequesters it in the cytoplasm, inhibiting its transcriptional activity (Schiaffino et al., 2013). In the absence of such inhibition, FOXO1 can induce transcription of Atrogin‐1 which is an E3 ubiquitin ligase that causes atrophy via increased protein degradation (Leger et al., 2006; Sandri et al., 2004). Thus, it is possible that the improvements in muscle healing caused by LIV are mediated by Akt through the inhibition of FOXO1 and Atrogin‐1 activity.

To determine if LIV signals are directly transduced by muscle cells, we applied LIV to differentiating C2C12 mouse myoblasts in culture. Our data show that LIV significantly increased myotube differentiation and diameter after 6 days of treatment. Similar to its effects in muscle injury, LIV increased total levels of Akt early during LIV treatment, and phosphorylated Akt as well as the ratio of phosphorylated‐to‐total Akt at a later time point. However, in cultured myotubes, increased Akt activity was not associated with reduced FOXO1 or Atrogin‐1 expression. In fact, expression of genes related to muscle regeneration and atrophy were largely unaffected by LIV; only MyoD was increased at day 6. Thus, other genes may mediate the LIV‐induced increase in myotube differentiation and size and/or the panel of genes assessed may be altered at other time points. Taken together, our data indicate that muscle cells are capable of directly transducing LIV signals into enhanced differentiation and growth, and Akt may be involved in this process.

In our previous study, we found similar effects of whole‐body vibration using LIV signals of 0.2 g applied at 90 Hz or 0.4 g applied at 45 Hz (Corbiere et al., 2017). These previous data indicate that signals within this range provide similar healing benefit and, thus, we chose the 0.2 g/90 Hz signals for this study. In addition, we have generated data using a skin wound healing model comparing 0.3 g and 1.0 g accelerations and have found that the 0.3 g signals improve healing, whereas the 1.0 g signals impair healing, indicating that signals with large accelerations may be detrimental to healing (our unpubl. data). Thus, high‐amplitude signals may impair tissue repair.

Previous studies have tested the effects of vibration on cultured myoblasts, and muscle of humans and mice, albeit with different vibration protocols (Ceccarelli et al., 2014; Chang, Lin, Yang, & Yang, 2018; Wang et al., 2010). Numerous studies have explored the effects of higher intensity vibration as an exercise stimulus on healthy, intact muscle with a focus on neuromuscular outcomes (Alam et al., 2018). These reports varied significantly in experimental design as well as in their main findings (Alam et al., 2018). Wang et al. (2010) applied vibration at 5, 8, and 10 Hz with an amplitude of 0.4 mm (compared to <0.1 mm in our study) for 10 min per day for up to 6 days to confluent C2C12 myoblasts in growth medium and observed an increase in myotube number, size, and fusion. These measurements corresponded with an increase in gene expression of MyoD and myogenin; both markers of myogenic differentiation. This study differed from ours in that LIV was applied while cells were in growth medium supplemented with 10% FBS rather than differentiation medium supplemented with 2% HS. Furthermore, although the vibration parameters used would be classified as “low‐intensity”, the frequencies used were lower and the amplitude was higher than those used in our study. In another study, Ceccarelli et al applied vibration in vivo to developing mice and in vitro to primary myoblasts (Ceccarelli et al., 2014). Vibration was applied at 30 Hz and 11 mm amplitude for 1 hr per day over a period of 7 days. This vibration signal would not be classified as LIV due to the increased intensity with such an amplitude. This higher‐intensity vibration showed an increase in cell fusion when applied directly to cells in culture (Ceccarelli et al., 2014). This study also found a significant increased expression of M‐cadherin, an indicator of satellite cell activation, in mouse TA muscles, primary myoblasts, C2C12 myoblasts, and L6C11 myoblasts when treated with vibration. Further studies have investigated the effects of LIV on muscle in aging individuals in humans and mice. Three months of whole‐body vibration in older adults with sarcopenia increased skeletal muscle mass index (Chang et al., 2018), however, 6 months of local vibration in postmenopausal women had no effect on muscle mass in the quadriceps (Tankisheva et al., 2015). Lastly, vibration decreased damage from pressure ulcers and associated oxidative damage in senescence‐accelerated mice, but not controls (Wong et al., 2017). The differences in the outcomes and mechanisms implicated in these studies may be due to the differences in study design and vibration parameters. Despite these differences, it appears that there is a range of vibration signals that can induce myogenesis or growth of muscle fibers in vitro and in vivo.

Although our data demonstrate that LIV acts directly on muscle cells, LIV may also have effects on other cells involved in muscle repair. One potential reason that healing from traumatic muscle injury is impaired compared with other types of muscle injury is the disruption of blood supply. LIV has been shown to improve skin wound healing in diabetic mice, associated with new blood vessel formation (Weinheimer‐Haus et al., 2014). LIV can also increase blood flow in the skin of hairless mice, the lower leg of healthy human subjects, and the forearm of both healthy subjects and those with Type 2 diabetes (Ichioka, Yokogawa, Nakagami, Sekiya, & Sanada, 2011; Lohman, Petrofsky, Maloney‐Hinds, Betts‐Schwab, & Thorpe, 2007; Maloney‐Hinds, Petrofsky, Zimmerman, & Hessinger, 2009). Furthermore, LIV can improve healing of pressure ulcers in humans in part via nitric oxide–mediated increases in blood flow (Arashi et al., 2010). Thus, it is possible that LIV also improves muscle healing via increased neoangiogenesis and blood flow; such a potential link deserves further study.

Our study has several limitations. Although we assessed potential mechanisms in a number of ways, the data are essentially correlative. Further experiments using pharmacological or genetic approaches to target factors or pathways are necessary to demonstrate their role in LIV‐induced healing. In addition, the response of cells grown in culture likely depends on growing conditions, notably substrate stiffness. We grew cells directly on cell culture plastic, which is much stiffer than normal biological substrates and responses to LIV may differ on softer substrates. Regardless, we saw remarkably similar effects of LIV in culture as we did in mice. We observed some modest changes in mRNA expression in response to LIV, however, whether these translate into changes in protein expression important for muscle repair remains to be determined. Furthermore, we have not yet identified the optimal signals for inducing muscle repair, the characteristics of the signals that promote repair, or the mechanotransduction machinery involved in cells or tissues. A final limitation is that we did not assess functional characteristics related to recovery. We plan to investigate these issues in a future study.

In summary, our findings are consistent with our hypothesis that local LIV improves muscle regeneration following injury and myotube growth and differentiation in culture; however, this does not appear to be mediated by IGF‐1. Muscle cells were able to directly transduce LIV signals into growth and differentiation, which was associated with increased Akt signaling. As Akt plays a central role in maintaining muscle mass and works through multiple pathways, future studies could be focused on further elucidating the role of Akt in LIV‐induced muscle repair.

## CONFLICT OF INTEREST

TJK has a patent pending regarding the application of vibrations for therapeutic treatment. TC certifies that he, or a member of his or her immediate family, has no funding or commercial associations (e.g., consultancies, stock ownership, equity interest, patent/licensing arrangements, etc.) that might pose a conflict of interest in connection with the submitted article.

## AUTHOR CONTRIBUTIONS

TC performed the experiments. TK and TC conceived and designed the experiments, analyzed the data, and wrote the article.
